# Metabolomics reveals citric acid secretion in mechanically–stimulated osteocytes is inhibited by high glucose

**DOI:** 10.1038/s41598-018-38154-6

**Published:** 2019-02-19

**Authors:** Alma Villaseñor, Daniel Aedo-Martín, David Obeso, Igor Erjavec, Juan Rodríguez-Coira, Irene Buendía, Juan Antonio Ardura, Coral Barbas, Arancha R. Gortazar

**Affiliations:** 1 IMMA, Institute of Applied Molecular Medicine, School of Medicine, CEU San Pablo University, Campus Monteprincipe, Boadilla del Monte, 28668 Madrid, Spain; 2 Basic Medical Sciences Department, School of Medicine, CEU San Pablo University, Campus Monteprincipe, Boadilla del Monte, 28668 Madrid, Spain; 3 Centre for Metabolomics and Bioanalysis (CEMBIO), Faculty of Pharmacy, CEU San Pablo University, Campus Monteprincipe, Boadilla del Monte, 28668 Madrid, Spain; 4 Laboratory for Mineralized Tissues, Center for Translational and Clinical Research, School of Medicine, University of Zagreb, Zagreb, Croatia

## Abstract

Osteocytes are the main cells of bone tissue and play a crucial role in bone formation and resorption. Recent studies have indicated that Diabetes Mellitus (DM) affects bone mass and potentially causes higher bone fracture risk. Previous work on osteocyte cell cultures has demonstrated that mechanotransduction is impaired after culture under diabetic pre-conditioning with high glucose (HG), specifically osteoclast recruitment and differentiation. The aim of this study was to analyze the extracellular metabolic changes of osteocytes regarding two conditions: pre-conditioning to either basal levels of glucose (B), mannitol (M) or HG cell media, and mechanical stimulation by fluid flow (FF) in contrast to static condition (SC). Secretomes were analyzed using Liquid Chromatography and Capillary Electrophoresis both coupled to Mass Spectrometry (LC-MS and CE-MS, respectively). Results showed the osteocyte profile was very similar under SC, regardless of their pre-conditioning treatment, while, after FF stimulation, secretomes followed different metabolic signatures depending on the pre-conditioning treatment. An important increment of citrate pointed out that osteocytes release citrate outside of the cell to induce osteoblast activation, while HG environment impaired FF effect. This study demonstrates for the first time that osteocytes increase citrate excretion under mechanical stimulation, and that HG environment impaired this effect.

## Introduction

Mechanical loading is an important regulator of bone mass, by which our skeleton adapts to the changes in load by altering its mass, shape and microstructure^1^. In fact, an increase in mechanical loading is positively reflected in bone formation, as occurs during physical exercise. Meanwhile, reduced physical activity leads to bone loss by increasing osteoclast function, as occurs in immobilized patients or astronauts^2^. Accumulated evidence demonstrates that osteocytes are the main mechanosensors in bone. These cells, considered as orchestrators of bone remodeling, are trapped in the mineralized bone matrix inside the lacunae, and are connected among themselves and other cells on the bone surface through a network of dendritic processes. It has been described that osteocytes are able to control osteoblast function by producing sclerostin, which inhibits osteoblast activity by antagonizing the Wnt/β-catenin pathway^3–5^. Furthermore, osteocytes produce the ligand of the receptor activator for nuclear factor κB (RANKL) that supports osteoclast differentiation and activity^6^. However, a lack of mechanical loading induces osteocyte apoptosis, which is related to the recruitment and differentiation of osteoclast precursors^7,8^. The underlying mechanisms targeting bone resorption at specific bone sites are still not fully understood.

Diabetes Mellitus (DM) is a complex disease characterized by high levels of glucose (HG) in serum due to either lack of insulin production (type 1 DM) or malfunctions in its signaling (type 2 DM), with negative effects on several organs including the skeleton. Type 1 DM causes a decrease in bone mass, whereas type 2 DM affects bone architecture as bone mass is usually normal or even increased; but in both cases, subjects with DM show a trend of higher bone fracture risk^9,10^. It has been demonstrated that a diabetic background affects osteocyte mechanotransduction by impairing several mechanisms induced through mechanical forces in both *in vivo* and *in vitro* models^11–13^. Recently, our group has shown that HG alters the secretome of mechanically stimulated osteocyte cells, affecting the manner these cells communicate with osteoclasts^14^.

Over recent years, metabolomics has been widely employed as a new approach to describe the metabolic phenotype in multifactorial and complex diseases such as DM^15–21^. Metabolomics, which works by measuring hundreds of metabolites as the representation of the metabolic condition, has recently been employed by Shum *et al*. to observe *in vivo* metabolic changes in aging bone comparing different mouse strains^22^. However, it has not yet been applied to the *in-vitro* study of the osteocyte secretome. Therefore, to investigate the effect of HG on mechanically stimulated osteocytes, we performed metabolomics analysis on cell culture media using a multiplatform approach including liquid chromatography and capillary electrophoresis, both coupled to mass spectrometry (LC-MS and CE-MS, respectively). With this approach, we managed to investigate the metabolic background of osteocyte communication and the effect of HG environment on osteocyte metabolic activity after mechanical stimulation. Our findings highlight citrate as a key metabolite in mechanically stimulated osteocytes, and that this process is impaired by the HG environment.

## Materials and Methods

### Experimental design

The osteocyte MLO-Y4 cell line, derived from murine long bones^23^, was supplied by Dr. Lynda Bonewald (University of Missouri, Kansas City, MO). Cells were grown in α-MEM cell media supplemented with 2.5% fetal bovine serum (FBS), 2.5% calf serum (CS) and 1% penicillin-streptomycin in 5% CO2 at 37 °C. Osteocytes were plated at 2 × 10^4^ cells/cm2 on glass slides (FlexCell, Hillsborough, NC) and after 24 hours of incubation these underwent two treatment stages. First, cells went through pre-conditioning, where fresh media with or without high D-glucose or mannitol as osmotic control (25 mM) were added for 24 h. These groups will be referred to as basal (B), high glucose (HG) and mannitol (M), respectively. Afterwards, all cell media were replaced by normal-glucose α-MEM cell medium. Half of the cells were then submitted to mechanical stress by laminar fluid flow (FF) with a shear stress of 10 dynes/cm2, 8 Hz, for 10 minutes in a Flexcell® Streamer® Shear Stress Device. The other half were kept at static conditions (SC). Finally, cells were cultured with fresh medium for additional 18 h to collect the cell medium (CM), which was immediately removed and kept to −80 °C until the day of analysis. No significant changes in cell viability were observed between the different experimental conditions (with or without HG and/or FF). The details of the experiment can be found in a previously published article^14^.

### Metabolite extraction

#### LC-MS sample treatment

CM samples were thawed on ice and mixed vigorously using vortex. From each sample, 200 μl were taken and placed in an Eppendorf tube. Then, 5 μL of acetonitrile (ACN) were added to later enhance metabolite recovery from filtration. Samples were then vortexed and placed on a Centrifree® ultra-centrifugation device with a cut-off porous of 30 kDa (Millipore Ireland Ltd., Cork, Ireland). In this way, deprotenization was carried out by centrifugation applying 2000 × *g*, 80 minutes at 4 °C. The filtrate was transferred directly into a LC vial.

#### CE-MS analysis

For this technique, prior to ultracentrifugation, 200 μl of each CM were mixed with 5 μL of ACN containing formic acid (FA; 4.1 mM) and methionine sulfone as internal standard (IS, 22.5 mM)^24,25^. After centrifugation (2000 × *g*, 80 minutes at 4 °C), the filtrate was taken into a CE vial.

#### Quality control preparation

Quality control (QC) sample was prepared by pooling equal volumes of CM samples. For each technique, an independent QC was prepared for each analytical platform. The QC sample was treated following the same procedure described above for samples. QC sample was analyzed throughout the worklist to provide evidence about the stability, performance and reproducibility of the analytical technique.

### Metabolic profile analysis

All cell media samples were prepared and analyzed in separate runs in a randomized order for the corresponding analytical technique.

#### Liquid Chromatography-Quadrupole Time of Flight-Mass Spectrometry (LC-QTOF/MS Analysis)

Samples were analyzed on a 1290 Infinity series UHPLC system coupled through an electrospray ionization (ESI) source with Jet Stream technology to a 6550 iFunnel QTOF/MS system (Agilent Technologies, Waldbronn, Germany). For the separation, an injection volume of 0.5 μL was introduced to a reversed-phase column (Zorbax Eclipse XDB-C18, 4.6 × 50 mm, 1.8 µm, Agilent Technologies) kept at 40 °C. The system was operated at 0.5 mL/min flow rate consisted of Solvent A, water with FA at 0.1%, and solvent B, methanol, as mobile phases. Gradient started with 2% B (0–5 min), later a linear gradient from 2 to 50% B (6–15 min), sustained at 50% B for 2 min (15–17 min), then up to 95% B (17–18 min), kept to 95% B for 2 min (18–20 min), and returned to starting conditions in 1 min to finally keep the re-equilibration with 2% B until 25 min in total. Detector was operated in full scan mode (m/z 100 to 1100) in positive and negative electrospray ionization (ESI) mode at a scan rate of 1 scan/s. Accurate mass measurement was assured through an automated calibrator delivery system that continuously introduced a reference solution, containing masses of *m/z* 121.0509 (purine) and *m/z* 922.0098 (HP-921) in positive ESI mode; whereas *m/z* 112.9856 (TFA) and *m/z* 922.009798 (HP-921) in negative ESI mode. The capillary voltage was 3500 V and 4000 V for positive and negative ionization mode, respectively. The nebulizer and gas flow rate were 35 psig and 11 L/min respectively. The fragmentor voltage was set to 75 V and the radiofrequency voltage in the octupole to 750 V (OCT RF Vpp)^26^.

#### Capillary Electrophoresis-Time of Flight-Mass Spectrometry (CE-TOF/MS Analysis)

The equipment consisted on a CE (7100 Agilent) coupled to a TOF/MS analyzer (6224 Agilent). Separation was carried out using a fused-silica capillary (total length of 100 cm; i.d. of 50 μm; Agilent technologies). The capillary was pre-conditioned with 1 M NaOH for 30 min, followed by MilliQ® water and background electrolyte-BGE (0.8 M of FA in 10% methanol) for 30 min, at 20 °C, using normal polarity. Before each analysis, the capillary was flushed for 5 min (950 mbar pressure) with BGE. The MS was operated in positive polarity, with a full scan from 80 to 1000 m/z at a rate of 1.4 scans/s. Drying gas was set to 10 L/min, nebulizer to 10 psi, voltage to 3.5 kV, fragmentor to 125 V, gas temperature to 200 °C and skimmer to 65 V. Sheath liquid composition was methanol/water (1:1, v/v) containing 1.0 mM of FA and two reference masses which allowed mass correction and higher mass accuracy *m/z* 121.0509 (purine) and *m/z* 922.0098 (HP-921). Samples were injected at 50 mbar for 50 s and stacked by applying background electrolyte at 100 mbar for 10 s. The separation voltage was 30 kV with 25 mbar of internal pressure and a total running time of 30 min^24,25^.

### Data treatment

Acquired data were processed using MassHunter Profinder (B.07.00, Agilent Technologies) software to obtain a structured data matrix in an appropriate format. Raw data were analyzed through two algorithms, both applied consecutively. First, molecular feature extraction (MFE) algorithm, which reduces the data size and complexity by removing associated non-specific information and extracting important variables (features). Secondly, for obtaining better accuracy of the data, find by ion (FbI) algorithm was used to perform a targeted feature extraction. Finally, abundance of the molecule, mass and retention time for each feature in all samples was obtained in a matrix data form. The quality of the data collected by LC-MS and CE-MS was assured excluding background noises and unrelated ions by keeping molecular features present in the 50% of QC injections with a coefficient of variation (CV) below 30%, and present in 75% of the samples of the study. Missing values were estimated using k-nearest neighbors (kNN) algorithm.

### Metabolite Identification

Tentative identification (TI) was performed for all significant compounds, while identity (ID) confirmation was done either on those significant compounds with a fold change (FC) above 2 or below −2, or selecting those with a reliable TI hit from the databases. TI was performed for both techniques by searching accurate masses against public online databases – KEGG, Metlin, LipidMaps and HMDB – through an engine in-house web tool called CEU Mass Mediator^27^. For LC-MS, identification was confirmed by LC-MS/MS experiments on the same instrument as the first analyses (QTOF 6550 iFunnel MS, Agilent Technologies) using the same initial chromatographic conditions. Ions were targeted based on previously determined mass-charge and retention time, nitrogen was used as the collision gas, and fragmentation energy was set at 20 V. If available, the MS/MS spectra were compared against the MS/MS spectra from an online library (METLIN). For compounds whose fragmentation pattern was not present in the METLIN database, the patterns were predicted using ACD/ ChemSketch software v.12.01 (ACD/Labs, Toronto, ON, Canada). When possible, final confirmation of the identity was performed using commercially available standards. For CE-MS, the identity of the compound was assessed by spiking the commercial standard to the sample and checking the increment of the signal compared against sample and standard alone. Additionally, pathway analysis for the main comparisons were carried out using MetaboAnalyst 4.0 (www.metaboanalyst.ca).

### ATP Determination

ATP was measured with a ATP determination kit (Molecular Probes), following the instructions of the manufacturer^28^. Cells were lysed with 1× Passive Lysis Buffer (Promega) and were diluted 1:10 using the ATP determination kit reactant mix leading to a total volume of 100 μl. Luminescence was determined directly after the addition of the lysate to the reaction, and was quantified in a Luminometer (Turner Designs, Sunnyvale, CA). ATP concentrations in experimental samples were calculated by using an ATP standard curve.

### Citrate estimation

Changes in citrate were measured using a modification of MassHunter Metabolomics Dynamic MRM Database and Method from Agilent (Application note)^29^. The samples were analyzed on an UHPLC (1290 Infinity, Agilent Technologies) coupled to an ESI (AJS)-QQQ-MS (6460, Agilent Technologies) mass spectrometer. Briefly, to 50 μL of each CM, 150 μL of cold methanol were added to achieve protein precipitation. Samples were vortex-mixed for 1 min, incubated on ice for 5 min and centrifuged at 15,400 × g for 20 min at 4 °C. Then, 150 μL of the supernatant were drawn and transferred to a vial with glass insert, which was kept in the auto sampler until injection^29^.

### Statistical analysis

Data were analyzed using multivariate analysis (MVA; SIMCA P + 14.0.1 software, Umetrics (Umea, Sweden)) and univariate statistics (UVA; MATLAB R2015a software (Mathworks, Inc., Natick, USA)). For MVA, log-transformation and Center scaling were used for LC-MS data whereas Center scaling was used for CE-MS data. For unsupervised modeling, principal components analysis (PCA) was used to confirm data quality, detect outliers and check sample patterns. Additionally, discriminant models such as partial least squares discriminant analysis (PLS-DA) and orthogonal PLS-DA (OPLS-DA) were performed to confirm the separation between groups. For UVA, non-parametric 2–way ANOVA test was used to obtain the significant signals, followed by Benjamini-Hochberg (FDR, false discovery rate) *post hoc* correction (q = 0.05). Moreover, pairwise comparisons were obtained using Mann−Whitney U tests. Statistical significance level was set with a 95% of confidence interval (p < 0.05). The percentage of change (%) was calculated as follows: [(average value in the case group − average value in reference group)/(average value in reference group)] × 100.

## Results

### Osteocyte secretome data quality

After a careful sample analysis and data treatment, the number of measured features in CM samples were 654 and 806 for LC-MS in positive and negative mode, respectively, and 160 features for CE-MS (Fig. S1A). The quality of the analysis was assessed in 2 ways: through the control graph of the signal intensity throughout the experiment, and through multivariate analysis (MVA) using a non-supervised model. The signal intensity of each CM profile reflected by their total useful signal (TUS) along the worklist showed that quality control (QC) injections were all in the same level for the three techniques (Fig. S1B). The clustering of QC injections in the PCA models – where, without prior information about the samples, the model clusters tightly the QCs in the center of the models – demonstrated the high quality of the data (Fig. S1C).

### Metabolic profile and Statistical analyses

**Figure 1:**
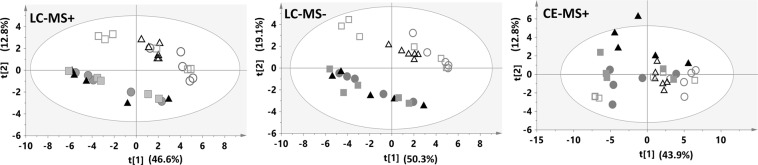
Non-supervised PCA plots generated using only samples for the three analytical techniques.Annotations: filled dot, BSC; filled square, MSC; filled triangle, HGSC; empty dot, BFF; empty square, MFF; and empty triangle, HGFF.

A second PCA model was performed after removing QCs (Fig. 1), in which we observed a separation of samples treated with FF (B-FF, HG-FF and M-FF) in respect to the clustering of SC (B-SC, HG-SC and M-SC) samples. This effect suggests that the mechanical stimulation exerts higher effect than high glucose (HG) preconditioning on the metabolic profile of the osteocytes in the LC-MS data. This confirmed that the main differences on the secretomes were a consequence of the FF stimuli.

Moreover, differences between groups were tested using PLS-DA and OPLS-DA discriminant analysis models between pairs of groups. Thus, Fig. S2 shows the cross-validated OPLS-DA plots between the SC groups and their corresponding FF group. Most models showed high predictive capacity having a Q^2^ quality value above 0.9; however, no valid models were obtained in any of the comparisons between the three SC groups. This means that without mechanical stimulation, HG pre-conditioning did not strongly affect the metabolic signature at SC. On the other hand, cross-validated OPLS-DA models between the three FF groups showed that osteocytes presented impaired metabolic processes (Fig. S3).

**Figure 2:**
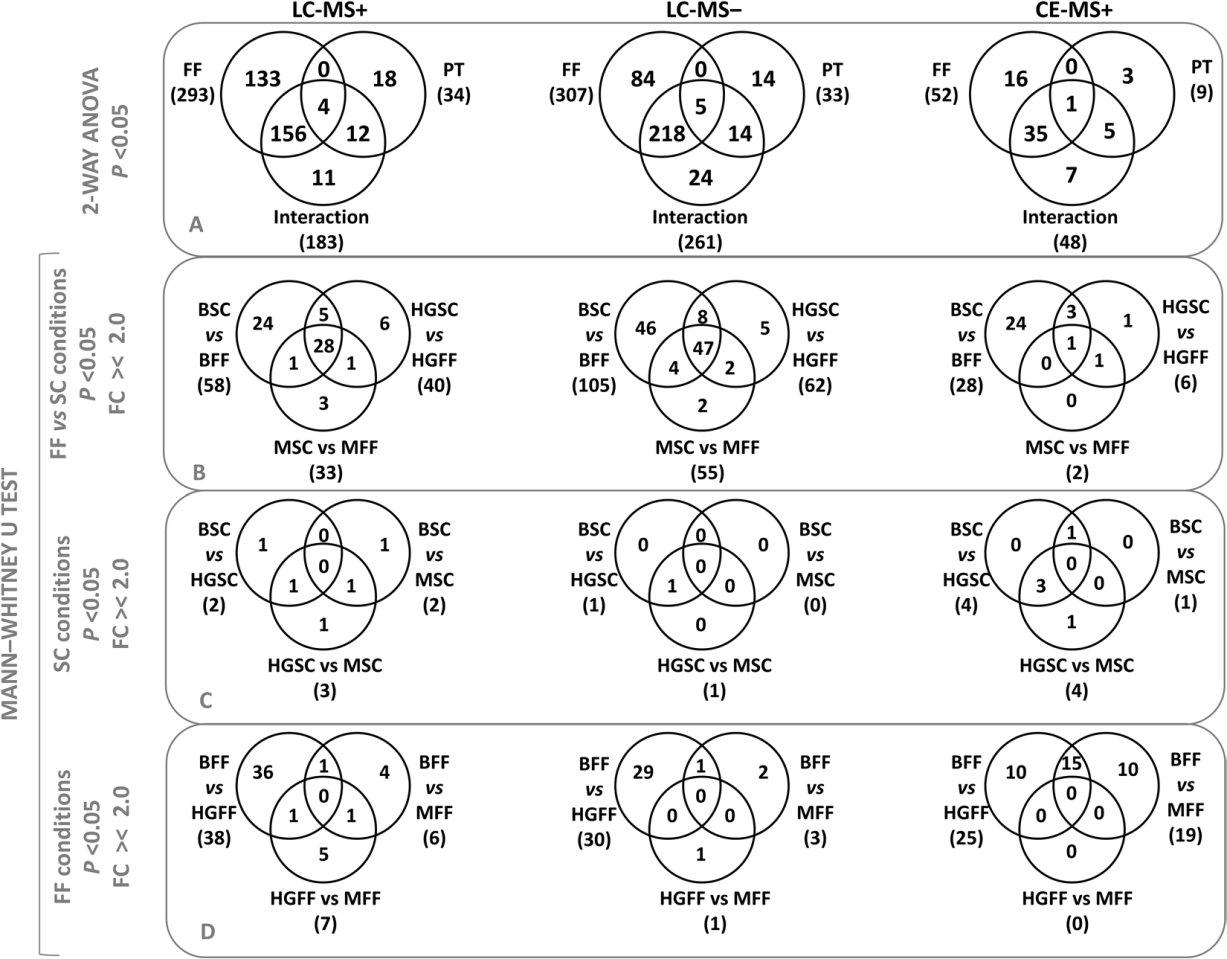
Venn diagrams for the three techniques: LC-MS+, LC-MS− and CE-MS. Significant variables (*p* value < 0.05) from the (**A**) non-parametric 2-way ANOVA, or after Mann-Whitney U test with a FC >/< 2.0, (**B**) between SC and FF conditions within each pre-treatment or (**C**) between the pre-treatment at SC and (**D**) after the FF stimuli. Key: PT, pre-treatment.

In addition, results from the univariate statistical analysis (UVA) using non-parametric 2-way ANOVA were represented in Venn diagrams for the three techniques (Fig. 2A). Here, the biggest number of significant variables was due to the FF factor (SC or FF) as opposed to cell media pretreatment (PT) factor (B, HG or M). Consecutively, pairs of groups were compared using the criteria of *p* < 0.05 and FC higher than 2 or lower than −2 (Fig. 2B–D). The results showed that the comparison of B-SC *vs* B-FF presented the largest number of changes. Furthermore, the effect of osteocyte PT was represented on Fig. 2C for SC, and Fig. 2D for FF groups. Regarding SC, there were barely no differences between the three groups. On the other hand, after FF stimuli, osteocyte PT had a distinctive effect on cell metabolism, with the highest number of differences being between HG-FF and B-FF groups. The differences shown on Fig. 2(B,D) were also projected onto Volcano plots (Fig. S4) showing the direction of the changes.

**Figure 3:**
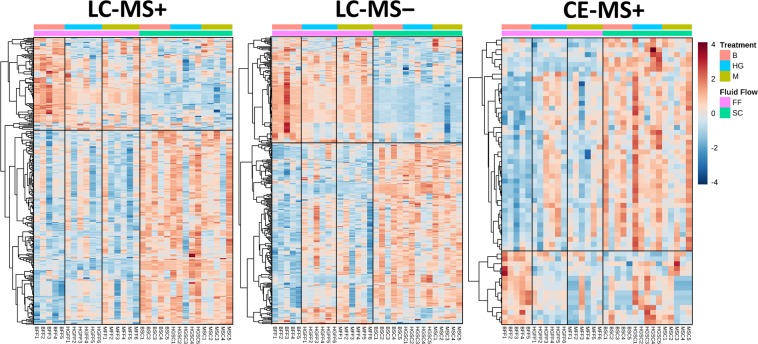
Heat maps for LC-MS+, LC-MS− and CE-MS. Significant variables (*p* value < 0.05) from the non-parametric 2-way ANOVA.

Consequently, these differences were visually represented using heat maps (Fig. 3). The heat maps from the three techniques showed clear differences between SC and FF conditions and pointed out a different metabolic signature depending on the pre-treatment after the FF test. Specifically, the HG-FF medium presented a distinctive metabolic pattern. This pattern was not observed in neither of the control conditions (B-FF and M-FF). Moreover, results showed that in in the three techniques there were a higher number of metabolites whose levels decreased on the CM after FF stage compared to SC conditions.

### Metabolite identification and pathway analysis

Regarding identification, 101 and 123 masses were selected for MS/MS fragmentation in LC‒MS + and LC-MS−, respectively. In addition, 20 commercial standards were spiked to samples and confirmed for CE-MS. The list of identified metabolites was summarized on Table S1, with a total of 51 metabolites. From the list: amino acids, pyrimidine and purine metabolites, carboxylic acids, vitamins and dipeptides were identified. Moreover, their percentage of change (%) between the groups was annotated on Table S2.

**Figure 4:**
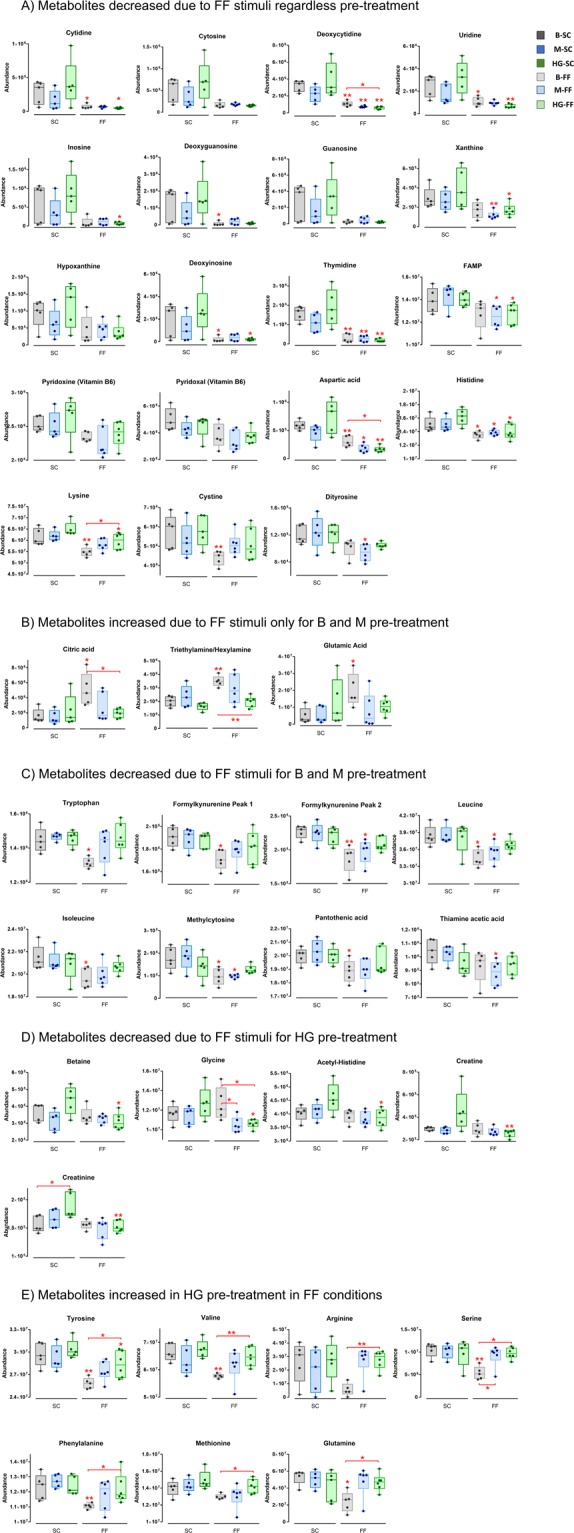
Representative plots of significant metabolites in the different groups. Key: *means *p* < 0.05 and **p < 0.01.

Regarding the effect of the FF stimuli over SC, the majority of identified metabolites in the CM were significantly decreased. Consistent changes, regardless of the PT, affected purine and pyrimidine degradation products, vitamin B6 and its degradation products, amino acids and Krebs cycle intermediates (Fig. 4A). These metabolites included: cytidine, cytosine, deoxycytidine, uridine, inosine, deoxyguanosine, guanosine, xanthine, hypoxanthine, deoxyinosine, thymidine, FAMP, vitamin B6 (pyridoxine and pyridoxal), aspartate, histidine and lysine. The changes in purine and pyrimidine metabolites were higher in “HG-FF *vs* HG-SC” compared to the other comparisons.

Surprisingly, citrate was found increased over 200% ‒ in B-FF compared to B-SC‒ and 83% ‒ in M-FF compared to M-SC – in the cell medium. However, when comparing HG groups, citrate showed no significant change (Fig. 4B). Changes in citrate concentration in the different groups were checked with a different method and using an independent technique (LC-QQQ-MS) to discard any possible influence of matrix effect and these results were fully confirmed.

**Figure 5:**
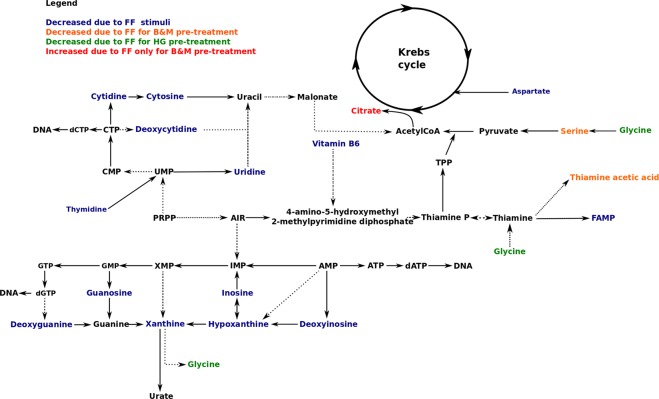
Major enriched metabolic pathway. Solid lines represent a direct reaction whereas spotted lines represent an indirect reaction.

From the list of metabolites (Tables S1 and S2), some were decreased in B-FF and M-FF compared to B-SC and M-SC, respectively, but not in HG groups. These included: tryptophan (Trp) and its degradation product formylkynurenine (FKN), leucine, isoleucine, methylcytosine, pantothenic acid, serine, phenylalanine and thiamine acetic acid (TAA) (Fig. 4C). On the other hand, some changes were significant only between HG groups (Fig. 4D), such as betaine, glycine, acetyl histidine, creatine and creatinine. These metabolites were all significantly decreased (28, 16, 16, 44 and 19%, respectively) in HG-FF. Moreover, in the case of SC groups, the results showed that only creatinine was significantly increased (21%) in HG-SC compared to B-SC. Finally, in the case of FF groups, B-FF showed differences compared to HG-FF. The latter group presented decreased levels of citrate, aspartate, deoxycytidine and glycine compared to the former (Fig. 4), while glutamine, tyrosine, valine, phenylalanine, tryptophan, serine and arginine were increased in HG-FF (Fig. 4E). Arginine, in particular, presented a notable increment of 396%. The main findings were depicted into a metabolic pathway in Fig. 5.

Moreover, intracellular ATP was determined in the MLO-Y4 protein extract. We observed a decrease in the intracellular ATP levels in B-FF and M-FF samples compared to B-SC and M-SC, respectively, but not in the HG samples (Fig. S5). This increase matched with the citrate production in these conditions.

In addition, pathway analyses were plotted for the main comparisons (Fig. S5). The parameters from the analysis are compared on Table 3S. The pathways with the highest impact factors were the biosynthesis of aromatic and branch-chained amino acids (AAA and BCAA, respectively) and the metabolism of glycine, serine and threonine.

## Discussion

Previous studies of our group have shown that HG affects osteocyte mechanotransduction, especially osteocyte-osteoclast communication, suggesting new pathways to link the mechanisms involved in the deleterious effects of DM in the skeleton^14^. We emphasized that the analysis of the metabolic secretome may be critical to obtain new insights into this process and the role of HG levels in these effects. This is the first time that metabolite extraction from cell media using ultracentrifugation through Centrefree with a 30 kDa cut-off diameter is analyzed by LC-MS. By following this methodology, the analysis of an almost intact sample is possible. The use of LC-MS and CE-MS enriched the metabolic signature of each secretome.

In the present study, we have observed an increased secretion of citrate in FF-stimulated osteocytes. Regarding citrate, this organic tricarboxylic acid is mainly synthesized from glycolisys-derived acetyl-CoA and oxalacetate, being a key intermediate in Krebs cycle. In most mammalian cells citrate is oxidized to generate ATP, but some cells have developed metabolic modifications to achieve net citrate production by sacrificing ATP production. This mechanism is based on preventing citrate entry into the oxidative phase of the Krebs cycle^30^. Previous studies have shown that citrate is concentrated in bone tissues, being an essential part of the hydroxyapatite crystal structure^31^. Citrate increases calcium binding in bone and hydroxyapatite crystal thickening, thus being determinant in bone stability and resistance to fracture^31^. Our data showing osteocyte secretion of citrate to the cell medium suggests that these cells may be specialized on citrate production instead of oxidizing it into the Krebs cycle. Moreover, osteocytes comprise 90–95% of the total bone cells, being the most abundant and long-lived cells in skeletal tissues and the orchestrators of the bone remodeling process^32^. Therefore, osteocytes most likely are the bone cells responsible for stabilization of hydroxyapatite crystals in bone.

On the other hand, our data demonstrate that extracellular amino acids are decreased in osteocytes after mechanical stimulus. From a metabolic point of view, the use of amino acids for energy is limited to situations in which the intake of regular sources of energy is insufficient to supply necessary ATP. Moreover, it is well known that mechanical stimulation causes a rapid (few minutes) and transient release of ATP^33^, so a possible explanation for these results could be that FF-stimulated osteocytes require amino acids as alternative energy sources to compensate decreased ATP production during oxidation arrest for citrate secretion, although further studies are needed to completely clarify this.

We also observed that HG inhibits FF-induced citric acid production and amino acid consumption (glutamine, tyrosine, valine, arginine, serine, phenylalanine and methionine) in osteocytes. The mechanisms responsible of these effects are still unknown. Some changes in the extracellular concentration of metabolites induced by HG in our study (i.e. valine, tyrosine, methionine) seem to be due to a mechanism independent of high osmotic pressure, given that mannitol acts as a control group in these situations. However, osmotic pressure actions should not be completely dismissed considering mannitol affects the extracellular concentration of other metabolites (i.e. arginine, serine and glutamine). It has previously been shown that osteoblasts, the cell precursors of osteocytes, appear to be more sensitive to extracellular hypertonicity than to the intracellular metabolic effects of hyperglycemia^34^. In this study, mannitol induced similar effects as HG on organic matrix production suggesting an extracellular hyperosmolarity effect in diabetic models *in vitro*^34^. Aside from extracellular hyperosmolarity, alternative effects of HG on cells have been previously described to be exerted through five major mechanisms: increased flux of glucose through the polyol pathway (causing intracellular osmotic stress), increased intracellular formation of advanced glycation end-products (AGEs), increased expression of the receptor for advanced glycation end products and its activating ligands, activation of protein kinase C (PKC) isoforms and over-activity of the hexosamine pathway^35^. All these seem to be activated by mitochondrial overproduction of reactive oxygen species (ROS)^35^. It has been described that in bone, accumulation of AGEs and ROS compromise matrix properties and potentially alter the function of osteocytes^36^. In this regard, we have previously shown that HG alters osteocyte secretion of factors that recruit osteoclasts, the matrix resorbing cells^14^. Further studies are needed to elucidate which of the aforementioned mechanisms mediate HG actions on the metabolome of osteocytes and to identify the molecular targets that may be altered by these mechanisms.

In addition, HG-preconditioning induces the secretion of other metabolites – namely betaine, glycine, acetyl-histidine, creatine and creatinine (a breakdown product of creatine) – before their consumption after FF process. Betaine administration has previously been shown to enhance insulin sensitivity in diet-induced-obese mice^37^ and increases insulin signaling pathways in isolated adipocytes^38^. Thus, it is possible that under SC, HG induces osteocyte secretion of betaine to improve extracellular glucose-removal actions of insulin in bone cells. However, FF decreases betaine secretion in the presence of HG. Betaine has also been described as an osmolyte that protects cells from environmental stress and is a catabolic source of methyl groups via transmethylation for use in many biochemical pathways^39^. Exposition of osteocytes to both extracellular HG concentrations and FF may require elevated intracellular levels of betaine to avoid glucose-derived extracellular hyperosmolarity.

Moreover, pathway analyses pointed out that AAA and BCAA pathways could be implicated in the mechanostransduction process and their impairment due to HG and hyperosmolarity. Interestingly, AAA and BCAA metabolic pathways have been largely described to be involved in bone turnover process through calcium sensor receptor (CaSR)^40–42^. The authors indicated that, whereas early markers of osteoclast differentiation are downregulated by AAA, indicating the inhibition of bone resorption, the BCAA pathway has the opposite effect causing a reduction in serum levels of growth factor (IGF-1)^40,43^.

In summary, we showed that osteocyte metabolic profile did not significantly change after mannitol and high glucose pre-treatment without mechanical stimulation. However, mechanical stimulation influenced osteocytes behavior as they became metabolically more active. In the cell medium we detected compounds involved in nucleic acid metabolism, vitamin B6 metabolism and citrate production. The high glucose environment completely inhibited increase in citrate production caused by mechanical stimulation. These findings suggest that osteocytes produce citrate when mechanically stimulated, and that this could be a key factor in bone remodeling and a new pathway affected in Diabetic Mellitus that could contribute to the deleterious effect of hyperglycemia.

## Supplementary information

Supplementary Information

## Data Availability

The data that support the findings of this study are available from the corresponding authors A.R.G. and A.V., upon request.
