# Supplementary material for: Metabolomics reveals citric acid secretion in mechanically–stimulated osteocytes is inhibited by high glucose
Source: Sci Rep. 2019 Feb 19;9:2295. doi: 10.1038/s41598-018-38154-6 (PMC6381120; doi:10.1038/s41598-018-38154-6)
Supplement: Supplementary file 1 — Supplementary Information [file 41598_2018_38154_MOESM1_ESM.docx]

**SUPPLEMENTARY INFORMATION**

**Metabolomics Reveals Citric Acid Secretion in Mechanically-Stimulated Osteocytes is Inhibited by High Glucose**

Alma Villaseñor^1*^, Daniel Aedo-Martín^1^, David Obeso^1, 3^, Igor Erjavec^4^, Juan Rodríguez-Coira^1^, Irene Buendía^1^, Juan Antonio Ardura^1,2^, Coral Barbas^3^, Arancha R. Gortazar^1,2*^. 31,42

**Figure S1.**

**Fig S1. Metabolic profile and quality data by LC-MS (positive and negative) and CE-MS.**

(A) Extracted Compound Chromatogram (ECC) from LC-MS +ESI mode, LC-MS –ESI mode and CE-MS +ESI mode; (B) Instrument performance represented by TUS *vs* injection order; (C) PCA plots generated using all samples plus QC injections.

Annotations: (
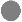
) means samples and (
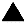
) QC injections.

**Figure S2.**

**Fig S2. OPLSDA cross-validated plots between pairs comparing SC *vs* FF for all techniques.**

Annotations: Q^2^ means the prediction capacity of the model; R^2^ means the sample variation explained by the model; (
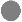
) BSC; (
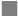
) MSC; (
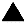
) HGSC; (
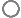
) BFF; (
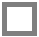
) MFF and (
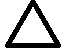
) HGFF.

**Figure S3.**

**Fig S3. OPLSDA cross-validated plots between pairs comparing B, M & HG, after FF**.

Annotations: Q^2^ means the prediction capacity of the model; R^2^ means the sample variation explained by the model; (
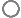
) BFF; (
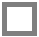
) MFF and (
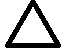
) HGFF.

**Figure S4.**

**Fig S4. Volcano plots for the three techniques: LC-MS+, LC-MS‒ and CE-MS.**

A) Significant variables (*p* value< 0.05) from the Mann-Whitney U test with a │FC│> 2.0, between SC and FF conditions within each CM pre-treatment, B) between the CM pre-treatment groups after the FF stimuli.

**Figure S5**

**Fig S5. Intracellular ATP levels.**

Annotations: *, stands for *p* value <0.05 between each pair of SC vs FF

**Figure S6.**

**Fig S6. Pathway analysis for the four main comparisons.**

A) Fluid Flow (FF) *vs* Static Conditions (SC); B) Basal FF (B-FF) *vs* High Glucose FF (HG-FF); C) B-FF *vs* Mannitol FF (M-FF) and D) M-FF *vs* HG-FF. Annotations: Pathway analysis carried out in MetaboAnalyst 4.0, Pathway library *Mus musculus* (KEGG), Pathway analysis algorithms were “Global Test” for Pathway Enrichment Analysis and “Relative-betweeness Centrality” for Pathway Topology Analysis

**Table S1. Significant metabolite identification from the three platforms; LC-MS+, LC-MS‒ and CE-MS.**

|  |  |  |  |  |  |  |  |  |  | **Factor1** | | **Factor2** | |  | |
| --- | --- | --- | --- | --- | --- | --- | --- | --- | --- | --- | --- | --- | --- | --- | --- |
|  |  |  |  |  |  |  |  |  |  | **FLUID FLOW** | | **PRE-TREATMENT** | | **INTERACTION** | |
| **No** | **Technique** | **Compound** | **m/z (Da)** | **Mass (Da)** | **RT/MT (min)** | **Error (ppm)** | **Adduct** | **Formula** | **Biological role** | ***p* value** | ***q* value** | ***p* value** | ***q* value** | ***p* value** | ***q* value** |
| 1^$^ | CE-MS+ | *Arginine | 174.1115 | 175.1188 | 11.65 | -1 | M+H | C6H14N4O2 | Amino acid | 0.533 | 0.623 | 0.258 | 0.551 | 0.149 | 0.231 |
| 2 | CE-MS+ | Aspartic acid | 134.0447 | 133.0375 | 16.68 | -0.4 | M+H | C4H7NO4 | Amino acid | 0.000 | 0.001 | 0.257 | 0.551 | 0.000 | 0.020 |
| 3 | CE-MS+ | Betaine | 118.0859 | 117.0787 | 16.54 | -2.2 | M+H | C5H11NO2 | Amino acid | 0.022 | 0.078 | 0.869 | 0.883 | 0.119 | 0.207 |
| 4 | LC-MS+ | Carnitine | 162.1133 | 161.1052 | 0.91 | 0.0 | M+H | C7H15NO3 | Quaternary ammonium salts | 0.011 | 0.035 | 0.461 | 0.864 | 0.123 | 0.290 |
| 5 | LC-MS+ | Choline | 104.1076 | 103.0997 | 0.89 | -6.1 | M+ | C5H13NO | Quaternary ammonium salts | 0.009 | 0.029 | 0.403 | 0.864 | 0.102 | 0.264 |
| 6 | LC-MS- | Citric acid | 191.0200 | 192.0272 | 1.67 | 1.0 | M-H | C6H8O7 | Tricarboxylic acids | 0.0120 | 0.0437 | 0.3962 | 0.8850 | 0.0488 | 0.1522 |
| 7 | CE-MS+ | Creatine | 132.0777 | 131.0705 | 13.72 | 7.4 | M+H | C4H9N3O2 | Carboxylic acids | 0.012 | 0.048 | 0.542 | 0.667 | 0.055 | 0.136 |
| 8 | CE-MS+ | Creatinine | 114.0665 | 113.0593 | 11.72 | 3.4 | M+H | C4H7N3O | Amino acid | 0.012 | 0.048 | 0.479 | 0.652 | 0.033 | 0.105 |
| 9 | CE-MS+ | Cysteine | 241.0305 | 240.0233 | 16.26 | -2.3 | M+H | C6H12N2O4S2 | Amino acid | 0.011 | 0.048 | 0.715 | 0.768 | 0.062 | 0.149 |
| 10 | LC-MS+ | Cytidine | 244.0933 | 243.0852 | 1.26 | -1.3 | M+H | C9H13N3O5 | Pyrimidine nucleosides | 0.002 | 0.012 | 0.696 | 0.913 | 0.021 | 0.121 |
| 11 | LC-MS+ | Cytosine | 112.0505 | 111.0433 | 1.26 | 0.3 | M+H | C4H5N3O | Pyrimidines | 0.003 | 0.014 | 0.881 | 0.961 | 0.047 | 0.171 |
| 12 | LC-MS+ | Deoxycytidine | 112.0503 | 111.0432 | 1.49 | -0.6 | Frag | C9H13N3O4 | Pyrimidine nucleosides | 0.000 | 0.000 | 0.274 | 0.864 | 0.000 | 0.004 |
| 13 | LC-MS+ | Deoxyguanosine | 152.0571 | 151.0489 | 4.69 | 7.2 | Frag | C10H13N5O4 | Purine nucleosides | 0.002 | 0.009 | 0.749 | 0.928 | 0.029 | 0.149 |
| 14 | LC-MS+ | Deoxyinosine | 253.0931 | 252.0848 | 4.88 | -4.2 | M+H | C10H12N4O4 | Purine nucleosides | 0.001 | 0.006 | 0.716 | 0.913 | 0.010 | 0.086 |
| 15 | LC-MS- | Dityrosine | 359.1237 | 360.1309 | 4.94 | -3.4 | M-H | C18H20N2O6 | Lignans | 0.0005 | 0.0030 | 0.6374 | 0.9050 | 0.0185 | 0.0827 |
| 16 | LC-MS- | Folic acid | 440.1322 | 441.1394 | 11.06 | -0.6 | M-H | C19H19N7O6 | Carboxylic acids | 0.0299 | 0.0867 | 0.0807 | 0.8850 | 0.0284 | 0.1118 |
| 17 | CE-MS+ | Glucosamine-Tentative | 180.0880 | 179.0808 | 14.34 | 7.8 | M+H | C6H13NO5 | Carbohydrates | 0.002 | 0.018 | 0.386 | 0.607 | 0.028 | 0.096 |
| 18^$^ | CE-MS+ | Glutamic acid | 148.0590 | 147.0523 | 16.02 | -5.9 | M+H | C5H9NO4 | Amino acid | 0.136 | 0.246 | 0.182 | 0.551 | 0.095 | 0.187 |
| 19^$^ | CE-MS+ | Glutamine | 147.0751 | 146.0679 | 15.83 | -8.4 | M+H | C5H10N2O3 | Amino acid | 0.146 | 0.257 | 0.427 | 0.607 | 0.082 | 0.169 |
| 20 | CE-MS+ | Glycine | 76.0401 | 75.0329 | 13.00 | 11.6 | M+H | C2H5NO2 | Amino acid | 0.043 | 0.112 | 0.132 | 0.551 | 0.018 | 0.080 |
| 21 | LC-MS+ | Glycyl-Tyrosine Peak1 | 221.0928 | 238.0955 | 2.99 | 0.5 | M+H-H2O | C11H14N2O4 | Peptide | 0.047 | 0.106 | 0.826 | 0.953 | 0.287 | 0.427 |
| 22 | LC-MS- | Glycyl-Tyrosine peak2 | 219.0773 | 238.0951 | 2.08 | -1.1 | M-H-H2O | C11H14N2O4 | Peptide | 0.5087 | 0.6488 | 0.1162 | 0.8850 | 0.0453 | 0.1473 |
| 23 | LC-MS+ | Guanosine | 284.0993 | 283.0916 | 4.02 | -0.2 | M+H | C10H13N5O5 | Purine nucleosides | 0.007 | 0.026 | 0.958 | 0.988 | 0.142 | 0.318 |
| 24 | CE-MS+ | Histidine | 156.0767 | 155.0695 | 11.88 | 0.2 | M+H | C6H9N3O2 | Amino acid | 0.000 | 0.004 | 0.551 | 0.667 | 0.004 | 0.057 |
| 25 | LC-MS- | Inosine | 267.073 | 268.0802 | 4.03 | -2.1 | M-H | C10H12N4O5 | Purine nucleosides | 0.0021 | 0.0105 | 0.6950 | 0.9186 | 0.0459 | 0.1479 |
| 26 | LC-MS+ | Isoleucine | 132.1014 | 131.0943 | 2.19 | -2.5 | M+H | C6H13NO2 | Amino acid | 0.004 | 0.017 | 0.526 | 0.878 | 0.048 | 0.173 |
| 27 | LC-MS+ | Kynurenine | 192.0662 | 191.0574 | 4.12 | -2.6 | Frag | C11H12N2O4 | Organooxygen compounds | 0.355 | 0.518 | 0.381 | 0.864 | 0.235 | 0.392 |
| 28 | LC-MS+ | Leucine | 132.1023 | 131.0943 | 2.37 | -2.5 | M+H | C6H13NO2 | Amino acid | 0.000 | 0.003 | 0.588 | 0.899 | 0.009 | 0.076 |
| 29 | LC-MS- | Formylkynurenine | 235.072 | 236.0792 | 2.89 | -2.2 | M-H | C11H12N2O4 | Butyrophenones | 0.0001 | 0.0008 | 0.8661 | 0.9381 | 0.0028 | 0.0218 |
| 30 | LC-MS- | Formylkynurenine | 235.0721 | 236.0793 | 2.30 | -1.7 | M-H | C11H12N2O4 | Butyrophenones | 0.0069 | 0.0285 | 0.6564 | 0.9122 | 0.0660 | 0.1798 |
| 31 | LC-MS+ | Pyroglutamate | 130.0507 | 129.0424 | 1.77 | -1.3 | M+H | C5H9NO4 | Amino acid | 0.002 | 0.010 | 0.976 | 0.994 | 0.046 | 0.170 |
| 32 | CE-MS+ | Lysine | 147.1123 | 146.1051 | 11.25 | -2.9 | M+H | C6H14N2O2 | Amino acid | 0.000 | 0.009 | 0.092 | 0.551 | 0.002 | 0.057 |
| 33 | CE-MS+ | Methionine | 150.0573 | 149.0501 | 15.73 | -6.4 | M+H | C5H11NO2S | Amino acid | 0.015 | 0.058 | 0.053 | 0.551 | 0.026 | 0.096 |
| 34 | LC-MS+ | Methylcytosine | 126.0658 | 125.0585 | 1.01 | -3.3 | M+H | C5H7N3O | Diazines | 0.001 | 0.007 | 0.811 | 0.942 | 0.010 | 0.086 |
| 35 | LC-MS+ | N-Acetyl-L-Histidine | 198.0881 | 197.0799 | 2.96 | -0.7 | M+H | C8H11N3O3 | Carboxylic acids | 0.013 | 0.042 | 0.574 | 0.896 | 0.105 | 0.269 |
| 36 | LC-MS+ | N-Formyl-4-amino-5-aminomethyl-2-methylpyrimidine | 167.0933 | 166.0853 | 1.19 | -1.0 | M+H | C7H10N4O | Thiamine metabolism | 0.001 | 0.005 | 0.996 | 0.996 | 0.030 | 0.150 |
| 37 | LC-MS- | Pantothenic acid | 218.1037 | 219.1109 | 8.03 | 1.0 | M-H | C9H17NO5 | Carboxylic acids | 0.0015 | 0.0080 | 0.8659 | 0.9381 | 0.0434 | 0.1464 |
| 38 | LC-MS- | Phenylalanine | 164.0716 | 165.0788 | 4.96 | -1.1 | M-H | C9H11NO2 | Amino acid | 0.0085 | 0.034 | 0.1861 | 0.8850 | 0.0300 | 0.1145 |
| 39 | LC-MS+ | Pyridoxal (Vitamin B6) | 168.0663 | 167.0579 | 1.58 | -2.1 | M+H | C8H9NO3 | Vitamin | 0.002 | 0.011 | 0.362 | 0.864 | 0.046 | 0.170 |
| 40 | LC-MS+ | Pyridoxine (Vitamin B6) | 170.0819 | 169.0737 | 1.77 | -1.1 | M+H | C8H11NO3 | Vitamin | 0.040 | 0.092 | 0.963 | 0.988 | 0.484 | 0.586 |
| 41 | CE-MS+ | Serine | 106.0503 | 105.0431 | 14.89 | 4.8 | M+H | C3H7NO3 | Amino acid | 0.012 | 0.048 | 0.440 | 0.612 | 0.031 | 0.104 |
| 42 | LC-MS+ | Thiamine acetic acid | 278.0837 | 278.0837 | 8.25 | -0.2 | M+ | C12H14N4O2S | Thiamine metabolism | 0.022 | 0.060 | 0.430 | 0.864 | 0.080 | 0.230 |
| 43 | LC-MS- | Thymidine | 241.083 | 242.0902 | 7.17 | -0.3 | M-H | C10H14N2O5 | Pyrimidine nucleosides | 0.000 | 0.000 | 0.7893 | 0.9220 | 0.0002 | 0.0031 |
| 44 | LC-MS+ | Triethylamine/ Hexylamine | 102.1278 | 101.1203 | 1.43 | -1.5 | M+H | C6H15N | Tertiary / Primary amines | 0.020 | 0.056 | 0.026 | 0.831 | 0.012 | 0.092 |
| 45 | CE-MS+ | Tryptophan | 205.0968 | 204.0896 | 16.21 | -1.4 | M+H | C11H12N2O2 | Amino acid | 0.062 | 0.140 | 0.066 | 0.551 | 0.050 | 0.133 |
| 46 | CE-MS+ | Tyrosine | 182.0809 | 181.0737 | 16.50 | -1.1 | M+H | C9H11NO3 | Amino acid | 0.001 | 0.010 | 0.185 | 0.551 | 0.003 | 0.057 |
| 47 | LC-MS- | Tyrosyl-Alanine-Frag-peak1 | 233.0928 | 252.1106 | 15.24 | -1.6 | M-H-H2O | C12H16N2O4 | Peptide | 0.1358 | 0.264 | 0.5940 | 0.8855 | 0.0976 | 0.2293 |
| 48 | LC-MS- | Tyrosyl-Alanine-Frag-peak2 | 233.0928 | 252.1106 | 15.42 | -1.6 | M-H-H2O | C12H16N2O4 | Peptide | 0.0039 | 0.0171 | 0.9424 | 0.9702 | 0.0861 | 0.2104 |
| 49 | LC-MS- | Uridine | 243.0619 | 244.0691 | 2.23 | -1.8 | M-H | C9H12N2O6 | Pyrimidine nucleosides | 0.0000 | 0.0003 | 0.5475 | 0.8850 | 0.0006 | 0.0072 |
| 50 | CE-MS+ | Valine | 118.0859 | 117.0787 | 14.90 | -2.4 | M+H | C5H11NO2 | Amino acid | 0.020 | 0.072 | 0.147 | 0.551 | 0.018 | 0.080 |
| 51 | LC-MS+ | Xanthine | 153.0413 | 152.0330 | 1.99 | -2.8 | M+H | C5H4N4O2 | Imidazopyrimidines | 0.000 | 0.002 | 0.371 | 0.864 | 0.007 | 0.062 |

**NOTE:** Table cells in green color denoted significant metabolites with a *p<* 0.05. *ID confirmed with standard. ^$^Arginine, glutamic acid and glutamine was included due to their significance for the comparisons between pairs.

**Table 2S.** **Pairwise comparisons using Mann-Whitney test.**

|  |  | **Mechanical loading vs Static conditions** | | | | | | **Pre-treatment effect after Mechanical loading** | | | | | | **Pre-treatment effect after Static conditions** | | | | | | |
| --- | --- | --- | --- | --- | --- | --- | --- | --- | --- | --- | --- | --- | --- | --- | --- | --- | --- | --- | --- | --- |
|  |  | **BSC *vs* BFF** | | **MSC *vs* MFF** | | **HGSC *vs* HGFF** | | **BFF *vs* HGFF** | | **BFF *vs* MFF** | | **MFF *vs* HGFF** | | **BSC vs HGSC** | | | **MSC vs HGSC** | | **BSC vs MSC** | |
| **No** | **Compound** | **% BFF** | **p value** | **% MFF** | **p value** | **% HGFF** | **p value** | **% HGFF** | **p value** | **% MFF** | **p value** | **% HGFF** | **p value** | **% HGSC** | **p value** | **% HGSC** | | **p value** | **% MSC** | **p value** |
| 1 | *Arginine | -78.59 | 0.095 | 36.61 | 0.662 | 2.82 | 0.792 | 396.32 | 0.004 | -79.4 | 0.017 | 2.23 | 0.004 | 3.33 | 1.000 | 35.83 | | 0.914 | -23.93 | 0.69 |
| 2 | Aspartic acid | -47.82 | 0.008 | -60.64 | 0.017 | -77.57 | 0.004 | -45.42 | 0.017 | 70.37 | 0.052 | -7.01 | 0.017 | 26.98 | 0.548 | 63.20 | | 0.171 | -22.19 | 0.222 |
| 3 | Betaine | -13.95 | 0.222 | -4.40 | 0.931 | -28.91 | 0.030 | -1.94 | 1.000 | -3.25 | 0.931 | -5.13 | 1.000 | 18.70 | 0.310 | 27.58 | | 0.352 | -6.96 | 0.690 |
| 4 | Carnitine | -4.06 | 0.548 | -9.52 | 0.082 | -18.91 | 0.126 | 0.92 | 0.931 | 3.75 | 0.537 | 4.70 | 0.485 | 19.39 | 0.310 | 16.82 | | 0.310 | 2.20 | 0.690 |
| 5 | Choline | -7.17 | 0.095 | -6.00 | 0.177 | -2.40 | 0.126 | 4.93 | 0.126 | -0.52 | 0.662 | 4.38 | 0.132 | -0.20 | 0.421 | 0.53 | | 0.421 | -0.72 | 0.841 |
| 6 | Citric acid | 219.92 | 0.016 | 83.52 | 0.177 | -15.08 | 0.662 | -61.89 | 0.004 | 91.65 | 0.126 | -26.97 | 1.000 | 43.55 | 1.000 | 57.82 | | 0.841 | -9.04 | 0.690 |
| 7 | Creatine | -1.37 | 0.841 | -3.63 | 0.429 | -44.08 | 0.030 | -13.20 | 0.429 | 7.84 | 0.662 | -6.39 | 0.429 | 53.10 | 0.151 | 61.33 | | 0.114 | -5.10 | 0.690 |
| 8 | Creatinine | -0.43 | 1.000 | -10.36 | 0.329 | -19.72 | 0.004 | -2.24 | 0.537 | 4.43 | 0.931 | 2.09 | 0.537 | 21.24 | 0.032 | 13.99 | | 0.114 | 6.37 | 0.222 |
| 9 | Cysteine | -23.58 | 0.008 | -3.81 | 0.792 | -12.41 | 0.247 | 15.80 | 0.247 | -13.55 | 0.052 | 0.10 | 0.247 | 1.03 | 1.000 | 9.94 | | 0.762 | -8.10 | 0.421 |
| 10 | Cytidine | -75.17 | 0.032 | -58.15 | 0.429 | -88.04 | 0.030 | -27.96 | 0.329 | 2.47 | 0.429 | -26.19 | 0.041 | 49.49 | 1.000 | 158.15 | | 0.222 | -42.09 | 0.222 |
| 11 | Cytosine | -67.57 | 0.056 | -46.82 | 0.329 | -78.20 | 0.126 | -10.39 | 0.931 | -6.89 | 0.429 | -16.56 | 0.041 | 33.34 | 1.000 | 103.57 | | 0.310 | -34.50 | 0.310 |
| 12 | Deoxycytidine | -66.63 | 0.008 | -66.93 | 0.004 | -84.21 | 0.004 | -42.01 | 0.030 | 45.40 | 0.052 | -15.69 | 0.394 | 22.54 | 1.000 | 76.57 | | 0.222 | -30.60 | 0.151 |
| 13 | Deoxyguanosine | -94.44 | 0.032 | -69.87 | 0.247 | -94.49 | 0.082 | 30.96 | 0.126 | -65.05 | 0.329 | -54.23 | 0.818 | 31.99 | 0.841 | 150.08 | | 0.310 | -47.22 | 0.421 |
| 14 | Deoxyinosine | -94.56 | 0.024 | -68.38 | 0.329 | -96.46 | 0.030 | -2.84 | 0.100 | -69.63 | 0.039 | -70.49 | 0.240 | 49.24 | 1.000 | 163.47 | | 0.222 | -43.36 | 0.690 |
| 15 | Dityrosine | -15.45 | 0.095 | -24.70 | 0.030 | -14.19 | 0.126 | 2.92 | 0.662 | 8.62 | 0.177 | 11.79 | 0.180 | 1.41 | 0.841 | -1.91 | | 0.841 | 3.38 | 0.690 |
| 16 | Folic acid | -7.07 | 0.056 | -6.53 | 0.126 | -0.10 | 0.792 | 9.41 | 0.017 | -0.43 | 0.792 | 8.94 | 0.065 | 1.78 | 0.690 | 1.92 | | 0.690 | -0.14 | 0.841 |
| 17 | Glucosamine-Tentative | -62.71 | 0.032 | -32.89 | 0.082 | -38.76 | 0.126 | 118.77 | 0.030 | -42.55 | 0.177 | 25.69 | 0.030 | 33.20 | 0.841 | 37.74 | | 0.762 | -3.29 | 0.841 |
| 18 | Glutamic acid | 301.31 | 0.016 | 39.09 | 0.931 | -18.51 | 0.792 | -48.24 | 0.082 | -60.79 | 0.082 | 31.99 | 0.082 | 154.90 | 0.310 | 125.30 | | 0.762 | 13.14 | 1.000 |
| 19 | Glutamine | -55.15 | 0.016 | -4.87 | 1.000 | 9.80 | 1.000 | 95.21 | 0.009 | 100.57 | 0.052 | -2.67 | 0.485 | -20.27 | 0.310 | -15.68 | | 0.914 | -5.44 | 0.548 |
| 20 | Glycine | 8.40 | 0.548 | -8.28 | 0.082 | -16.59 | 0.017 | -16.02 | 0.017 | 20.40 | 0.030 | 1.10 | 0.017 | 9.13 | 0.310 | 11.17 | | 0.476 | -1.84 | 0.841 |
| 21 | Glycyl-Tyrosine Peak1 | -9.70 | 0.016 | -8.72 | 0.247 | -1.41 | 0.792 | 6.97 | 0.329 | -1.41 | 0.931 | 5.46 | 0.310 | -2.03 | 0.548 | -2.36 | | 0.690 | 0.34 | 0.841 |
| 22 | Glycyl-Tyrosine peak2 | -21.13 | 0.032 | -10.00 | 0.662 | 12.97 | 0.247 | 36.50 | 0.004 | -16.05 | 0.126 | 14.59 | 0.093 | -4.70 | 0.690 | -8.70 | | 0.421 | 4.38 | 0.690 |
| 23 | Guanosine | -91.18 | 0.151 | -69.20 | 0.329 | -93.76 | 0.082 | -13.51 | 0.792 | -48.06 | 0.537 | -55.08 | 0.589 | 22.24 | 0.841 | 121.67 | | 0.310 | -44.85 | 0.548 |
| 24 | Histidine | -10.22 | 0.016 | -7.56 | 0.030 | -13.94 | 0.017 | 3.12 | 0.792 | -2.49 | 0.329 | 0.56 | 0.792 | 7.58 | 0.151 | 8.02 | | 0.610 | -0.40 | 1.000 |
| 25 | Inosine | -85.96 | 0.056 | -67.58 | 0.247 | -92.04 | 0.017 | -23.15 | 0.429 | -21.95 | 0.662 | -40.02 | 0.818 | 35.49 | 0.841 | 144.28 | | 0.222 | -44.53 | 0.690 |
| 26 | Isoleucine | -8.27 | 0.032 | -5.79 | 0.052 | -1.26 | 0.247 | 5.14 | 0.082 | -1.35 | 0.792 | 3.72 | 0.093 | -2.32 | 1.000 | -1.03 | | 0.548 | -1.30 | 0.690 |
| 27 | Kynurenine | 6.41 | 0.310 | -2.44 | 0.662 | -8.09 | 0.030 | -11.95 | 0.030 | 9.02 | 0.329 | -4.00 | 0.818 | 1.94 | 0.690 | 1.90 | | 1.000 | 0.04 | 0.548 |
| 28 | Leucine | -10.33 | 0.016 | -7.85 | 0.030 | -3.55 | 0.177 | 6.77 | 0.052 | -3.01 | 0.329 | 3.55 | 0.132 | -0.74 | 0.690 | -1.06 | | 0.690 | 0.33 | 1.000 |
| 29 | Formylkynurenine | -17.46 | 0.008 | -12.16 | 0.030 | -5.54 | 0.177 | 11.43 | 0.030 | -5.30 | 0.329 | 5.53 | 0.310 | -2.64 | 0.421 | -1.88 | | 1.000 | -0.77 | 0.690 |
| 30 | Formylkynurenine | -10.43 | 0.032 | -5.85 | 0.177 | -3.21 | 0.792 | 6.14 | 0.247 | -4.19 | 0.126 | 1.69 | 0.589 | -1.78 | 0.841 | -1.08 | | 0.548 | -0.71 | 0.841 |
| 31 | Pyroglutamate | -29.75 | 0.008 | -16.38 | 0.247 | -15.47 | 0.662 | 14.19 | 0.429 | -10.40 | 0.429 | 2.32 | 0.818 | -5.09 | 1.000 | 1.22 | | 1.000 | -6.24 | 0.310 |
| 32 | Lysine | -10.78 | 0.008 | -6.20 | 0.052 | -8.20 | 0.017 | 8.74 | 0.017 | -5.62 | 0.126 | 2.63 | 0.017 | 5.68 | 0.310 | 4.87 | | 0.476 | 0.77 | 0.548 |
| 33 | Methionine | -7.19 | 0.151 | -8.68 | 0.126 | -4.71 | 0.329 | 9.54 | 0.017 | -0.64 | 0.429 | 8.84 | 0.017 | 6.69 | 0.222 | 4.30 | | 0.914 | 2.29 | 0.841 |
| 34 | Methylcytosine | -45.48 | 0.032 | -47.57 | 0.030 | -9.29 | 0.429 | 32.43 | 0.329 | -1.24 | 1.000 | 30.78 | 0.002 | -20.42 | 0.310 | -24.41 | | 0.310 | 5.28 | 0.841 |
| 35 | N-Acetyl-L-Histidine | -2.32 | 0.690 | -7.07 | 0.247 | -16.05 | 0.030 | -2.11 | 0.931 | 2.98 | 0.537 | 0.81 | 1.000 | 13.91 | 0.095 | 11.60 | | 0.222 | 2.07 | 0.690 |
| 36 | N-Formyl-4-amino-5-aminomethyl-2-methylpyrimidine | -8.45 | 0.222 | -13.16 | 0.030 | -8.78 | 0.030 | 0.25 | 0.792 | 2.14 | 0.537 | 2.39 | 0.699 | 0.61 | 0.841 | -2.52 | | 0.310 | 3.21 | 0.690 |
| 37 | Pantothenic acid | -5.70 | 0.048 | -6.82 | 0.056 | -2.25 | 0.255 | 3.66 | 0.424 | -0.39 | 0.892 | 3.26 | 0.554 | 0.00 | 0.817 | -1.57 | | 0.524 | 1.60 | 0.524 |
| 38 | Phenylalanine | -10.37 | 0.008 | -7.10 | 0.139 | -1.77 | 0.329 | 10.31 | 0.009 | -6.40 | 0.312 | 3.24 | 0.792 | 0.65 | 0.794 | -2.36 | | 0.333 | 3.08 | 0.595 |
| 39 | Pyridoxal (Vitamin B6) | -23.89 | 0.151 | -21.83 | 0.126 | -17.24 | 0.126 | -1.47 | 1.000 | 13.43 | 0.429 | 11.76 | 0.240 | -9.38 | 1.000 | 5.57 | | 0.548 | -14.16 | 0.222 |
| 40 | Pyridoxine (Vitamin B6) | -4.85 | 0.151 | -3.14 | 0.329 | -4.99 | 0.329 | 0.35 | 0.931 | -0.90 | 0.662 | -0.56 | 1.000 | 0.51 | 0.548 | 1.39 | | 0.690 | -0.87 | 0.690 |
| 41 | Serine | -48.97 | 0.008 | -9.37 | 0.537 | -0.41 | 0.429 | 82.32 | 0.004 | -41.93 | 0.030 | 5.87 | 0.004 | -6.59 | 1.000 | -3.66 | | 0.610 | -3.04 | 0.690 |
| 42 | Thiamine acetic acid | -10.42 | 0.222 | -14.12 | 0.030 | -0.70 | 1.000 | 0.72 | 0.931 | 6.12 | 0.329 | 6.89 | 0.240 | -9.14 | 0.151 | -7.56 | | 0.222 | -1.71 | 0.841 |
| 43 | Thymidine | -81.30 | 0.008 | -75.15 | 0.004 | -90.24 | 0.004 | -37.78 | 0.931 | 9.83 | 0.792 | -31.66 | 0.589 | 19.24 | 0.841 | 74.03 | | 0.151 | -31.48 | 0.151 |
| 44 | Triethylamine/Hexylamine | 73.58 | 0.008 | 23.03 | 0.429 | 21.96 | 0.177 | -43.07 | 0.004 | 20.19 | 0.329 | -31.58 | 0.180 | -18.98 | 0.222 | -30.97 | | 0.222 | 17.38 | 0.690 |
| 45 | Tryptophan | -9.08 | 0.008 | -3.68 | 0.662 | 0.04 | 0.931 | 11.07 | 0.009 | -7.03 | 0.126 | 3.27 | 0.009 | 0.95 | 0.548 | -0.57 | | 1.000 | 1.53 | 0.310 |
| 46 | Tyrosine | -12.99 | 0.008 | -6.09 | 0.126 | -6.61 | 0.177 | 9.79 | 0.017 | -5.86 | 0.030 | 3.35 | 0.017 | 2.28 | 0.548 | 3.92 | | 0.914 | -1.58 | 0.690 |
| 47 | Tyrosyl-Alanine-Frag-peak1 | 5.64 | 0.421 | -3.06 | 0.662 | 21.39 | 0.009 | 4.02 | 0.792 | 7.82 | 0.329 | 12.15 | 0.041 | -9.48 | 0.222 | -10.44 | | 0.151 | 1.07 | 0.841 |
| 48 | Tyrosyl-Alanine-Frag-peak2 | -2.27 | 0.310 | -4.79 | 0.126 | -5.79 | 0.030 | -1.33 | 0.662 | 0.66 | 0.931 | -0.68 | 0.818 | 2.35 | 0.548 | 0.37 | | 0.690 | 1.98 | 0.421 |
| 49 | Uridine | -56.89 | 0.032 | -44.77 | 0.082 | -78.74 | 0.004 | -37.36 | 0.052 | 12.60 | 0.931 | -29.46 | 0.026 | 27.03 | 0.548 | 83.27 | | 0.151 | -30.69 | 0.151 |
| 50 | Valine | -13.20 | 0.008 | -2.67 | 1.000 | -4.02 | 0.329 | 12.25 | 0.004 | -6.54 | 0.082 | 4.91 | 0.004 | 1.51 | 0.841 | 6.38 | | 0.762 | -4.58 | 0.421 |
| 51 | Xanthine | -41.50 | 0.095 | -56.05 | 0.009 | -57.41 | 0.030 | -1.71 | 1.000 | 45.09 | 0.329 | 42.61 | 0.180 | 35.00 | 0.690 | 47.15 | | 0.421 | -8.26 | 0.690 |

**NOTE:** Table cells in red color denoted significant metabolites with a *p<* 0.05 whereas values in blue color were used to describe decrease and red color increase. *ID confirmed with standard. % means the change of one out of the two groups for each comparison.

**Table 3S.** **Pathway Analysis Parameters Comparisons.**

|  |  | **Comparisons** | | | | | | | | | | | | | |  |
| --- | --- | --- | --- | --- | --- | --- | --- | --- | --- | --- | --- | --- | --- | --- | --- | --- |
|  |  | **FF vs SC** | | | **BFF vs HGFF** | | | **BFF vs MFF** | | | | **MFF vs HGFF** | | | |  |
| **No** | **Pathway Name** | **Match Status** | **-log(p)** | **Impact** | **Match Status** | **-log(p)** | **Impact** | | **Match Status** | **-log(p)** | **Impact** | | **Match Status** | **-log(p)** | **Impact** | |
| 1 | Histidine metabolism | 2/15 | 17.4 | 0.24 | 1/15 | 4.7 | 0.00 | | ─ | ─ | ─ | | 1/15 | 0.2 | 0.00 | |
| 2 | Alanine, aspartate and glutamate metabolism | 1/24 | 15.0 | 0.19 | 1/24 | 4.7 | 0.19 | | ─ | ─ | ─ | | 1/24 | 0.2 | 0.19 | |
| 3 | Pyrimidine metabolism | 4/41 | 12.9 | 0.08 | 1/41 | 4.4 | 0.00 | | ─ | ─ | ─ | | 2/41 | 3.8 | 0.02 | |
| 4 | Aminoacyl-tRNA biosynthesis | 12/69 | 11.3 | 0.13 | 10/69 | 7.5 | 0.13 | | 4/69 | 4.9 | 0.13 | | 9/69 | 0.3 | 0.13 | |
| 5 | Arginine and proline metabolism | 2/44 | 10.1 | 0.01 | 2/44 | 5.2 | 0.08 | | 1/44 | 3.5 | 0.08 | | 2/44 | 0.2 | 0.08 | |
| 6 | Purine metabolism | 6/68 | 9.9 | 0.06 | ─ | ─ | ─ | | 1/68 | 3.2 | 0.00 | | ─ | ─ | ─ | |
| 7 | Tyrosine metabolism | 1/44 | 9.0 | 0.14 | 1/44 | 4.3 | 0.14 | | 1/44 | 3.3 | 0.14 | | 1/44 | 1.1 | 0.14 | |
| 8 | Phenylalanine metabolism | 2/11 | 8.7 | 0.41 | 2/11 | 5.1 | 0.41 | | 1/11 | 3.3 | 0.00 | | 1/11 | 1.1 | 0.00 | |
| 9 | Phenylalanine, tyrosine and tryptophan biosynthesis | 2/4 | 8.7 | 1.00 | 2/4 | 5.1 | 1.00 | | 1/4 | 3.3 | 0.50 | | 1/4 | 1.1 | 0.50 | |
| 10 | Valine, leucine and isoleucine biosynthesis | 3/11 | 7.2 | 1.00 | 1/11 | 6.6 | 0.33 | | ─ | ─ | ─ | | 1/11 | 1.2 | 0.33 | |
| 11 | Glycine, serine and threonine metabolism | 6/31 | 6.4 | 0.51 | 2/31 | 6.2 | 0.51 | | 2/31 | 4.3 | 0.51 | | 2/31 | 0.5 | 0.51 | |
| 12 | Vitamin B6 metabolism | 2/9 | 6.2 | 0.57 | ─ | ─ | ─ | | ─ | ─ | ─ | | ─ | ─ | ─ | |
| 13 | Pantothenate and CoA biosynthesis | 3/15 | 7.6 | 0.02 | 1/15 | 6.6 | 0.00 | | ─ | ─ | ─ | | 1/15 | 1.2 | 0.00 | |
| 14 | Cysteine and methionine metabolism | 3/27 | 5.1 | 0.25 | 2/27 | 7.3 | 0.11 | | 1/27 | 4.1 | 0.03 | | 2/27 | 0.8 | 0.11 | |
| 15 | Glutathione metabolism | 2/26 | 4.7 | 0.01 | 1/26 | 4.2 | 0.01 | | 1/26 | 4.1 | 0.01 | | 1/26 | 0.3 | 0.01 | |
| 16 | Citrate cycle (TCA cycle) | 1/20 | 3.2 | 0.05 | 1/20 | 6.1 | 0.05 | | ─ | ─ | ─ | | ─ | ─ | ─ | |
| 17 | Glyoxylate and dicarboxylate metabolism | 1/18 | 3.2 | 0.26 | 1/18 | 6.1 | 0.26 | | ─ | ─ | ─ | | ─ | ─ | ─ | |
| 18 | Methane metabolism | 2/9 | 3.3 | 0.40 | 2/9 | 6.2 | 0.40 | | 2/9 | 4.3 | 0.40 | | 2/9 | 0.5 | 0.40 | |
| 19 | Glycerophospholipid metabolism | 1/30 | 2.9 | 0.02 | ─ | ─ | ─ | | ─ | ─ | ─ | | ─ | ─ | ─ | |
| 20 | Primary bile acid biosynthesis | 1/46 | 2.2 | 0.03 | 1/46 | 4.2 | 0.03 | | 1/46 | 4.1 | 0.03 | | 1/46 | 0.3 | 0.03 | |
| 21 | Tryptophan metabolism | ─ | ─ | ─ | 2/40 | 5.7 | 0.29 | | ─ | ─ | ─ | | 1/40 | 0.9 | 0.18 | |

Annotations: Pathway analysis carried out in MetaboAnalyst 4.0, Pathway library *Mus musculus* (KEGG), Pathway analysis algorithms were “Global Test” for Pathway Enrichment Analysis and “Relative-betweeness Centrality” for Pathway Topology Analysis.
